# Eclectic characterisation of chemically modified cell-derived matrices obtained by metabolic glycoengineering and re-assessment of commonly used methods†

**DOI:** 10.1039/d0ra06819e

**Published:** 2020-09-23

**Authors:** Silke Keller, Anke Liedek, Dalia Shendi, Monika Bach, Günter E. M. Tovar, Petra J. Kluger, Alexander Southan

**Affiliations:** Institute of Interfacial Process Engineering and Plasma Technology IGVP, University of Stuttgart Nobelstraße 12 70569 Stuttgart Germany alexander.southan@igvp.uni-stuttgart.de; Fraunhofer Institute for Interfacial Engineering and Biotechnology IGB Nobelstraße 12 70569 Stuttgart Germany; Department of Biomedical Engineering, Worcester Polytechnic Institute Worcester MA USA; School of Applied Chemistry, Reutlingen University Alteburgstraße 150 72762 Reutlingen Germany; University of Hohenheim, Core Facility, Module 3: Analytical Chemistry Unit Emil-Wolff-Str. 12 70599 Stuttgart Germany

## Abstract

Azide-bearing cell-derived extracellular matrices (“clickECMs”) have emerged as a highly exciting new class of biomaterials. They conserve substantial characteristics of the natural extracellular matrix (ECM) and offer simultaneously small abiotic functional groups that enable bioorthogonal bioconjugation reactions. Despite their attractiveness, investigation of their biomolecular composition is very challenging due to the insoluble and highly complex nature of cell-derived matrices (CDMs). Yet, thorough qualitative and quantitative analysis of the overall material composition, organisation, localisation, and distribution of typical ECM-specific biomolecules is essential for consistent advancement of CDMs and the understanding of the prospective functions of the developed biomaterial. In this study, we evaluated frequently used methods for the analysis of complex CDMs. Sodium dodecyl sulphate polyacrylamide gel electrophoresis (SDS-PAGE) and (immune)histochemical staining methods in combination with several microscopic techniques were found to be highly eligible. Commercially available colorimetric protein assays turned out to deliver inaccurate information on CDMs. In contrast, we determined the nitrogen content of CDMs by elementary analysis and converted it into total protein content using conversion factors which were calculated from matching amino acid compositions. The amount of insoluble collagens was assessed based on the hydroxyproline content. The Sircol™ assay was identified as a suitable method to quantify soluble collagens while the Blyscan™ assay was found to be well-suited for the quantification of sulphated glycosaminoglycans (sGAGs). Eventually, we propose a series of suitable methods to reliably characterise the biomolecular composition of fibroblast-derived clickECM.

## Abstract 2

Common characterisation methods for cell-derived extracellular matrices (ECMs) are compared using both unmodified and azide-bearing fibroblast-derived ECM.

## Graphical abstract

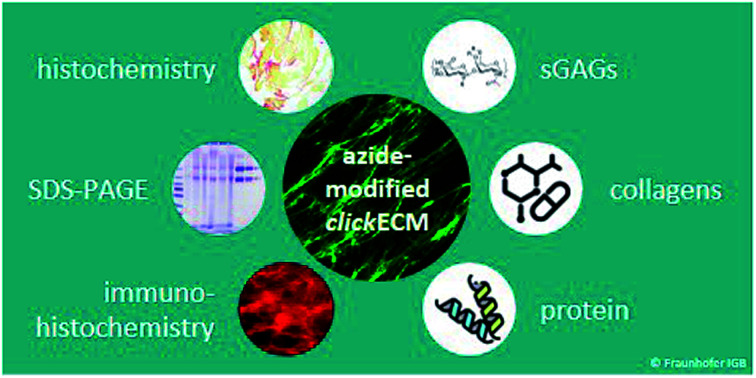

## 1. Introduction

Azide-bearing cell-derived extracellular matrices (“clickECMs”) have emerged as a very interesting class of biomaterials. These materials have been shown to combine functional properties of natural extracellular matrix (ECM) with the presence of metabolically incorporated small abiotic functional groups. *In vivo* but also applied as a biomaterial, the ECM is the natural, tissue-specific 3D-microenvironment of cells and regulates many crucial cellular functions such as cell adhesion, proliferation, migration or signal transduction.^1–4^

Azides are small abiotic groups which can be incorporated into cellular and extracellular biomolecules *via* metabolic glycoengineering (MGE). This technique includes the addition of chemically modified monosaccharides to the cell culture medium. During their natural metabolism, cells process the modified sugars and convert them into building blocks, which are then incorporated into both intra- as well as extracellular compounds.^5–9^

The first attempt to utilize MGE for the modification of natural cell-derived ECM with azide groups (–N_3_) was introduced by Ruff *et al.* in 2017.^10^ They performed MGE with the synthetic monosaccharide 1,3,4,6-tetra-*O*-acetyl-*N*-azidoacetyl-galactosamine (Ac_4_GalNAz) on primary human dermal fibroblasts to modify *O*-linked glycan structures with azide groups.^10–12^ These small chemical functional groups are able to undergo highly selective and specific bioorthogonal 1,3-dipolar Huisgen cycloadditions with alkyne groups. This reaction leads to a covalent attachment *via* triazole rings and can therefore be used for bioorthogonal bioconjugation.^13–17^

Using the same monosaccharide but adipose-derived stem cells (ASCs) instead of fibroblasts, Nellinger *et al.* demonstrated that this method can be used to generate azide-modified tissue-specific ECM.^18^ As an alternative to the above mentioned Ac_4_GalNAz, Gutmann *et al.* demonstrated the successful modification of NIH-3T3 fibroblasts-derived ECM using the glucosamine derivate 2-azidoacetylamino-2-deoxy-(1,3,4,6)-tetra-*O*-acetyl-d-glucopyranoside (Ac_4_GlcNAz).^19,20^

Despite the successful modification of ECM with azides *via* MGE and their demonstrated applicability as surface coatings or bioconjugation platforms,^10,17,19^ it is not yet understood if MGE changes properties of azide-modified ECM (*e.g.* the overall protein content or the biomolecular composition) beyond the modification with azide groups. Bioanalytical characterisation of these materials is distinctly challenging due to the insoluble and highly complex nature of cell-derived matrices.^17,21^ Yet, qualitative as well as quantitative analysis of the overall material composition, organisation, localisation, and distribution of typical ECM-specific biomolecules is essential for the course of biomaterial research in order to eventually understand and predict the prospective functions and performance of the developed biomaterial.

In this contribution, we re-assessed frequently used qualitative and quantitative bioanalytical methods suitable to reliably characterise the azide-modified clickECM in direct comparison with the unmodified fibroblast-derived ECM. For this, we applied commonly used qualitative and quantitative bioanalytical methods to investigate which one of them are able to deliver reliable results despite the highly complex and insoluble nature of ECM. We identified a set of appropriate methods to investigate the overall composition and distribution of typical ECM proteins as well as the architecture and the complexity of the matrices investigated in this study. Furthermore, we were able to estimate the total protein content, the contents of soluble and insoluble collagens as well as the amount of sulphated glycosaminoglycans of both unmodified ECM as well as azide-modified clickECM.

## 2. Experimental

### 2.1. Materials

A full list of all materials and instruments used in this study is provided in the ESI.†

### 2.2. Methods

#### 2.2.1. Cells

Primary fibroblasts were isolated from human foreskin obtained from three healthy volunteers (all under 1 year of age) under informed consent according to ethical approval granted by the ethical committee of the Landesärztekammer Baden-Württemberg (IGBZSF-2012-078). Isolation of fibroblasts was performed as previously described.^10,22^ In brief, cells were seeded in 175 cm^2^ tissue culture flasks in Dulbecco's Modified Eagle Medium (DMEM), supplemented with 10% fetal calf serum (FCS) and 1% penicillin/streptomycin (P/S). Cells were expanded until passage ten under standard cell culture conditions (5% CO_2_ and 95% humidity at 37 °C), collected by treatment with 0.05% trypsin–EDTA, and resuspended in supplemented DMEM until further use.

#### 2.2.2. Generation and isolation of (clickECM)

Fibroblast-derived matrices (both, azide-modified as well as unmodified, from now on referred to as (click)ECM when both types of ECM are discussed) were generated and isolated as previously described.^17^ In brief, cells were seeded in tissue culture polystyrene dishes (*Φ* 14.5 cm) at a density of 3.2 × 10^6^ cells per dish (20 000 cells per cm^2^) on day 1 and were then cultured for another seven days in DMEM supplemented with 10% FCS, 1% P/S, and 50 μg mL^−1^ Na-l-ascorbate. On day 5, cells were treated with either 625 μL 50 μM GalNAc (resulting in the unmodified ECM, “ECM”) or 625 μL 50 μM Ac_4_GalNAz per dish (resulting in the azide-modified “clickECM”). The medium was partly exchanged every two to three days (conditioned media change). To do so, half of the culture medium was carefully removed and replaced with fresh medium supplemented with Na-l-ascorbate and the respective sugar solution. On day eight, cells were lysed by washing three times with ultrapure water, followed by a treatment with 360 mM ammonia solution (NH_4_OH) for 15 minutes at 37 °C to isolate (click)ECM. This remaining (click)ECM was purified by three repeated washing steps with ultrapure water before it was stored at room temperature (RT) under sterile conditions until further use.

#### 2.2.3. Concentration and homogenisation of (click)ECM

After isolation through osmotic lysis, the isolated (click)ECM was concentrated and homogenised as previously described.^17^ In brief, concentration of isolated (click)ECM was achieved using sterile ultracentrifugation tubes with regenerated cellulose membranes (molecular weight cut-off (MWCO): 10 kDa). After centrifugation (4000 rpm for 90 minutes), concentrated (click)ECM was recovered and transferred into a microcentrifuge tube using a pipette.

Homogenisation of this concentrated (click)ECM was achieved using a bead mill and matching lysis tubes filled with ceramic beads and shaken in four intervals with homogenisation times of one minute per interval. Using a positive-displacement pipette, homogenised (click)ECM was then recovered.

#### 2.2.4. Sodium dodecyl sulphate polyacrylamide gel electrophoresis (SDS-PAGE) and Coomassie Brilliant Blue G-250 staining

Sodium dodecyl sulphate polyacrylamide gel electrophoresis (SDS-PAGE) was performed in order to compare the protein footprint of each separated (click)ECM sample. For this purpose, concentrated and homogenised (click)ECM aliquots were lyophilised and 40 μg dry (click)ECM portions were mixed with 40 μL ultrapure water and sonicated for 2 minutes (whereby sonication time was set to 10 seconds, followed by a 20 second break) at an amplitude of 60%. During sonication, samples were cooled on an ice bath. Prior to SDS-PAGE, sonicated samples were diluted in a 1 : 1 ratio with sample buffer for a final concentration of 0.5 mg mL^−1^. As reference, collagen type I was also diluted with ultrapure water to prepare solutions with 2 μg/40 μL, 6 μg/40 μL, and 10 μg/40 μL collagen concentrations which were then also diluted with sample buffer in the same 1 : 1 ratio for final concentrations of 0.025 mg mL^−1^, 0.075 mg mL^−1^, and 0.125 mg mL^−1^. Sample and reference proteins were denatured at 95 °C for 5 minutes. For electrophoresis, 5 μL of a pre-stained protein ladder was loaded together with the respective samples and collagen standards (40 μL each) onto a conventional commercially available 8–16% Tris–glycine polyacrylamide gradient gel. Separation was achieved by applying 225 V for 45–60 minutes.

After electrophoresis, the gel was equilibrated in ultrapure water for 20 minutes. Then, the gel was incubated in Imperial™ Protein Stain solution for 1.5 h on an orbital shaker. To remove excessive dye molecules, gels were subsequently washed in ultrapure water overnight. For evaluation of the band patterns, stained gels were placed into a plastic pocket and scanned with a conventional scanner.

#### 2.2.5. Histochemical analysis of cell-derived (click)ECM

Histochemical multichrome stainings (specifically Masson–Goldner trichrome, a modified Movat pentachrome, and Ladewig staining) were used to compare the overall (click)ECM composition and organisation. Stainings for individual ECM components (specifically Alcian blue-periodic acid-Schiff (PAS) and Picro Sirius Red staining) were performed to identify and compare glycosaminoglycans (GAGs) and collagens within the samples in a qualitative manner. Furthermore, an alkyne-coupled fluorophore was used to stain for azides within the azide-modified clickECM.

For these stainings, (click)ECM was isolated as described in 2.2.2, fixed with formalin for 18 h at RT, dehydrated with ascending ethanol concentrations (70%, 90%, 96%), isopropanol (100%), a 1 : 1 ratio mixture of 100% isopropanol – xylene and xylene solutions and subsequently embedded in paraffin. After embedding, sections of 5 μm thickness were cut using a microtome. The resulting sections were subsequently deparaffinised using RotiClear® and descending ethanol concentrations (96%, 70%, 50%, and deionised water).

Masson–Goldner-trichrome, Ladewig, and Alcian blue-PAS staining were carried out using the respective staining kits from Morphisto (Frankfurt am Main, Germany) according to the protocol provided by the manufacturer. The protocol for Movat staining was slightly modified by replacing the nucleus staining (Weigert solutions A and B) by a staining of elastic fibres with Weigert's resorcin–fuchsin staining solution (staining for 20 minutes at RT) as previously described.^10^ Picro Sirius Red staining was done by incubating deparaffinised sections in a staining solution composed of 0.1% Sirius Red in saturated aqueous picric acid for 1 hour.^23^ Incorporated azides within clickECM were stained using Alexa Fluor® 488-alkyne and the Click-iT® Cell Reaction Buffer Kit according to the manufacturer's instructions and described previously.^10,17^

With the exception of the sections used for the detection of azides within clickECM, which were washed twice with PBS^−^ prior to mounting with aqueous mounting medium and a glass cover slip, all other sections were desiccated by ascending ethanol concentrations (50%, 70%, and 96%), rinsed with isopropanol, and mounted with isomount mounting medium.

#### 2.2.6. Immunohistochemical analysis of cell-derived (click)ECM

Immunohistochemical analysis was done on (click)ECM generated in 35 mm ibidi-imaging dishes with a polymer coverslip bottom. For this purpose, 1.94 × 10^5^ fibroblasts in 300 μL medium were seeded into the depression and (click)ECM was generated as described in Section 2.2.2.

Decellularised (click)ECM was fixed in formalin for 10 minutes at RT and permeabilised by saponin (0.2% in PBS^−^) for 15 minutes. Samples were incubated with blocking solution (3% bovine serum albumin (BSA) + 0.1% Triton X-100 in PBS^−^) for 30 minutes at RT prior to staining to minimise nonspecific antibody protein interactions. Collagen type I, collagen type III, collagen type IV, fibronectin, and laminin of the proteins were then labelled with primary antibodies which were diluted in a 1 : 100 ratio with blocking solution for 1 hour at RT. Samples were washed three times with PBS^−^ and incubated for 1 hour in the dark at RT with the fluorophore-labelled secondary antibody solution (diluted in a 1 : 200 ratio in blocking solution). After washing three times with PBS-T (PBS^−^ supplemented with 0.1% Tween® 20), samples were washed once more with ultrapure water before covering the matrices with PBS^−^ prior to confocal laser scanning microscopy (cLSM). Matching isotype and secondary antibody controls were analysed in parallel to confirm the specificity of the primary antibodies.

#### 2.2.7. Quantification of the protein content of (click)ECM suspensions

##### 2.2.7.1. Bradford assay

The Bradford assay was carried out on sonicated (click)ECM samples using the Coomassie Plus-Assay-Kit from Thermo Fisher according to the manufacturer's instructions. For the ultrasonic treatment, 100 μL concentrated and homogenised (click)ECM were freeze-dried, mixed with 1 mL of water and sonicated for 6 min at 60% amplitude using an ultrasonic processor. To avoid thermal damage, samples were cooled on an ice bath during the process. As recommended by the manufacturer, BSA was used as a standard.

##### 2.2.7.2. BCA assay

The BCA assay was carried out on sonicated (click)ECM samples using the BCA assay from Pierce™ according to the manufacturer's instructions. Sonication was completed as described above (2.2.7.1). As recommended by the manufacturer, BSA was used as a standard.

##### 2.2.7.3. Elementary analysis of the nitrogen content for the estimation of the total protein content

For the estimation of the total protein content of insoluble (click)ECM suspensions, the nitrogen content was quantified *via* elementary analysis. For this purpose, the total nitrogen content of approximately 10 mg of the concentrated, homogenised, and lyophilised (click)ECM was determined following DIN EN ISO 16948 (“Solid biofuels – Determination of total content of carbon, hydrogen and nitrogen”) after dry combustion (elemental analysis). The analysis samples were burned in the oxygen stream at 900 °C. During oxidative combustion, molecular nitrogen and the oxidation products CO_2_, H_2_O, NO, NO_2_, SO_2_, SO_3_ were formed from the elements C, N and S. The resulting gas mixture was cleaned and separated into its components. The nitrogen oxides were quantitatively reduced to molecular nitrogen at the copper contact in the reduction tube and then determined relatively with an accuracy of up to ±0.1% using a thermal conductivity detector.

#### 2.2.8. Quantification of the collagen contents of (click)ECM

##### 2.2.8.1. Total collagen content *via* quantification and conversion of hydroxyproline (HP)

For this analysis, the protocol published by Capella-Monsonis *et al.*^24^ was slightly modified. For quantifying the hydroxyproline (HP) content, lyophilised (click)ECM samples (approximately 7 mg) were transferred into V-shaped borosilicate glass vials with polytetrafluoroethylene (PTFE) caps, diluted with 500 μL concentrated hydrochloric acid (HCl) and incubated overnight at 110 °C. After allowing the samples to cool down to RT, samples were carefully filtered through a PTFE syringe filter (*Φ* 0.2 μm) in order to remove the insoluble humin fraction.

For each sample, a dilution (50×) was prepared with ultrapure water. As reference samples, HP standard solutions of 0 μg mL^−1^ (blank), 1 μg mL^−1^, 2.5 μg mL^−1^, 5 μg mL^−1^, 10 μg mL^−1^, and 20 μg mL^−1^ were prepared with ultrapure water. Prior to the reaction, the assay diluent was prepared by mixing isopropanol and water in a 1 : 2 ratio. The citrate buffer was prepared by dissolving 17.19 g sodium acetate, 18.75 g tri-sodium citrate-dihydrate, and 2.75 g citric acid in 200 mL ultrapure water, which afterwards was mixed with 200 mL isopropanol and brought to a final volume of 500 mL with ultrapure water. Chloramine T and Ehlrich's solution were freshly prepared before each experiment. Chloramine T reagent was prepared by dissolving 0.2625 g chloramine T in 18.75 mL of the citrate buffer and addition of 3.75 mL ultrapure water. Ehrlich's reagent was prepared by dissolving 2 g 4-(dimethylamino)benzaldehyde (*p*-DMAB) in 3 mL 70% perchloric acid (HClO_4_) and 16.7 mL isopropanol were added.

For the reaction, 110 μL of the diluted samples or standards were mixed with 254 μL of the assay diluent and 176 μL of the freshly prepared chloramine T reagent in 1.5 mL-microcentrifugation tubes. The mixture was incubated for 10 minutes at RT. Next, 460 μL of freshly prepared Ehrlich's reagent were added to each microcentrifugation tube, mixed using a vortex mixer and incubated at 70 °C for 10 minutes. After incubation, 200 μL of each sample or standard were transferred into a 96-well plate and the absorbance of the solutions was measured at a wavelength of 555 nm against the blank samples using a spectrophotometer.

##### 2.2.8.2. Soluble collagen content *via* Sircol™ assay

The content of soluble collagens was quantified using the Sircol™ Soluble Collagen Assay from Biocolor according to the manufacturer's instructions. In brief, (click)ECM used for this experiment was generated in a 12-well plate with 7.76 × 10^4^ cells and 3 mL supplemented medium. Decellularisation and (click)ECM isolation was done as described in 2.2.2. Before collagen quantification, collagen-propeptides were extracted from the samples by adding 1 mL of a 0.1 mg mL^−1^ pepsin solution to each well and incubation at 4 °C for ∼16 hours. Supernatants were collected and supplemented with 100 μL neutralising reagent. These solutions were used for quantification of soluble collagens and all following steps were performed according to the manufacturer's instructions.

#### 2.2.9. Total sulphated glycosaminoglycan (sGAG) content

Total sulphated glycosaminoglycan (sGAG) content within (click)ECM samples was assessed using the Blyscan™ assay from Biocolor following the manufacturer's instructions with some modifications in sample preparation. (click)ECM used for this experiment was generated in a 12-well plate with 7.76 × 10^4^ cells and 3 mL supplemented medium. Decellularisation and (click)ECM isolation was completed as described in 2.2.2. Before sGAG quantification, sGAGs were extracted from the samples by adding 1 mL of a 0.1 mg mL^−1^ papain solution to each well and incubation at 65 °C for 3 hours. Supernatants were collected and centrifuged at 10 000*g* for 10 minutes. 500 μL of these solutions were used for sGAG quantification and all following steps were performed according to the manufacturer's instructions. Total sGAG contents were eventually calculated relative to the total (click)ECM dry mass of the used (click)ECM aliquots and are therefore expressed as mg sGAGs/mg (click)ECM.

#### 2.2.10. Statistical analysis

All experiments were completed in independently performed repeat attempts (*n* = 3) with (click)ECM from three individual donors. Measurements, unless stated otherwise, were run on triplicate samples.

Statistical significance was assessed by a one-tailed ANOVA and data was expressed as mean values ± standard deviation (s.d.). As labelled in the graphs, *p*-values lower than *α* = 0.05 (*), *α* = 0.01 (**), or *α* = 0.001 (***) were defined as statistically significant.

## 3. Results and discussion

Azide-modified clickECM emerged as a promising biomaterial combining the advantageous properties of natural cell-derived matrices (*e.g.* the outstanding bioactive properties) with abiotic functional groups which are a powerful tool for (bio)conjugation.^10,17–20^ Characterisation of the successful azide modification of cell-derived ECM *via* MGE was successfully completed by covalently linking clickECM azides to an alkyne-coupled fluorophore *via* Huisgen 1,3-dipolar azide–alkyne cycloaddition^10,18–20^ (ESI Fig. 1†). In contrast, characterising the material in terms of its biomolecular composition is not as trivial due to the highly complex biochemical composition, architecture, the high degree of cross-linking and thus its insolubility.^21,25–28^ Those characteristics make standard analytical methods comparatively difficult or not applicable. To identify reliable analytical methods to analyse the biomolecular composition of chemically modified clickECM beyond the modification with abiotic azide groups, we approached this aim by first applying qualitative analysis and comparison of the overall composition and organisation of unmodified ECM and azide-modified clickECM to investigate which methods are useful and reliable.

### 3.1. Qualitative assessment of clickECM composition

**Fig. 1:**
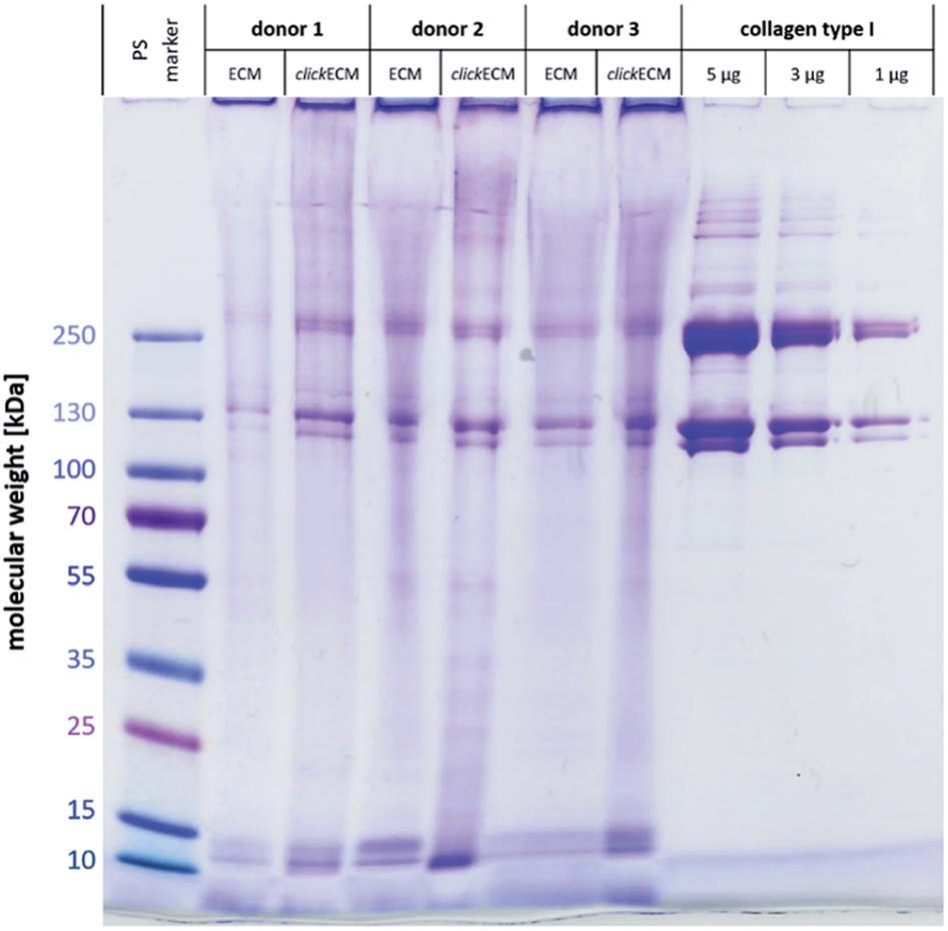
Representative image of a SDS-PAGE gel from *n* = 3 stained with Coomassie Brilliant Blue R-250 Imperial™ Protein Stain to examine and qualitatively compare the resulting protein footprint of electrophoretically separated (click)ECM and reference proteins.

Using SDS-PAGE and subsequent Coomassie staining, (click)ECM protein distribution was examined for three individual donors (Fig. 1). In addition, collagen type I was included as reference and a pre-stained protein marker was used to estimate the molecular weights of the separated proteins.

Gel electrophoresis and subsequent Coomassie Brilliant Blue R-250 Imperial™ Protein staining indicated a similar banding pattern for all separated (click)ECM samples regardless of the modification with azide groups *via* MGE or the donor. Dominant bands with a darker intensity were observed at approximately 10 kDa, 15 kDa, 120 kDa, 130 kDa, 250 kDa, and 260 kDa.

The bands at 10 kDa and 15 kDa can very likely be associated with small signal peptides as well as degradation products of protein cleavages. The darker intensity double bands at 120 kDa and 130 kDa refer to the lower molecular weight alpha regions and the darker intensity double bands at 250 kDa and 260 kDa correspond to the higher molecular weight cross-linked beta region in the collagen type I samples. As collagen accounts for up to 25% of total human protein, it can be considered the main component of ECM in general.^1,24^ The findings of this study are in close agreement with this fact as collagen was identified as a basic component in the *in vitro* generated (click)ECM in the SDS-PAGE banding pattern regardless of the azide modification.

The broader and more blurry looking bands very likely resulted from the high amount of glycoproteins within (click)ECM samples. As the levels of glycosylation within this class of ECM biomolecules is known to be quite different, the degree of SDS adsorption from the sample buffer may have contributed to blurry, uneven bands.^29^ Furthermore, and in direct comparison to collagen type I as a representative of a typical ECM-specific component, natural ECM consists of up to 300 different proteins in tissue-specific combinations and concentrations in a broad range of molecular weights also leading to additional less defined bands.^30,31^

The intensely stained gel pockets in the upper part of the gel suggest that there are also ECM components present, which are too large to penetrate the small-pored acrylamide gel. Another quite likely explanation for this effect could be that covalently cross-linked macromolecular complexes were present in the (click)ECM samples which is very likely as almost all main components of the ECM are known to exhibit peptide sequences that contribute to the cross-linking of the matrix.^1,32,33^ For this reason, the presence of a band in the SDS-PAGE gels can be used as a (qualitative) indication of the presence of a certain ECM component, but the absence of a band cannot be used as a proof for the absence of a component in the ECM sample.

Qualitatively, observations in SDS-PAGE for both ECM types and for all three donors were similar. The results of this first experiment did not suggest any impairment of the expression of ECM biomolecules through MGE. Moreover, the overall appearance of the complex banding patterns observed in this study was similar to the ones observed by Prewitz *et al.* who analysed ECM samples formed by mesenchymal stem cells (MSCs) under different stimuli.^34^ The same can be said for human glomerular ECM obtained from human placenta,^35^ human placenta-derived ECM sponges^36^ as well as cardiac and skeletal muscle ECM.^37^

In addition to the analysis of SDS-PAGE banding patterns, histological staining methods represent a well-established biochemical analysis tool that can be used for the visualisation of biological structures in the native, non-denatured state. As such, these methods facilitate the identification of various components as well as the analysis of their distribution within complex biological samples through the use of dyes, indicators as well as light and polarisation microscopy.^33^

**Fig. 2:**
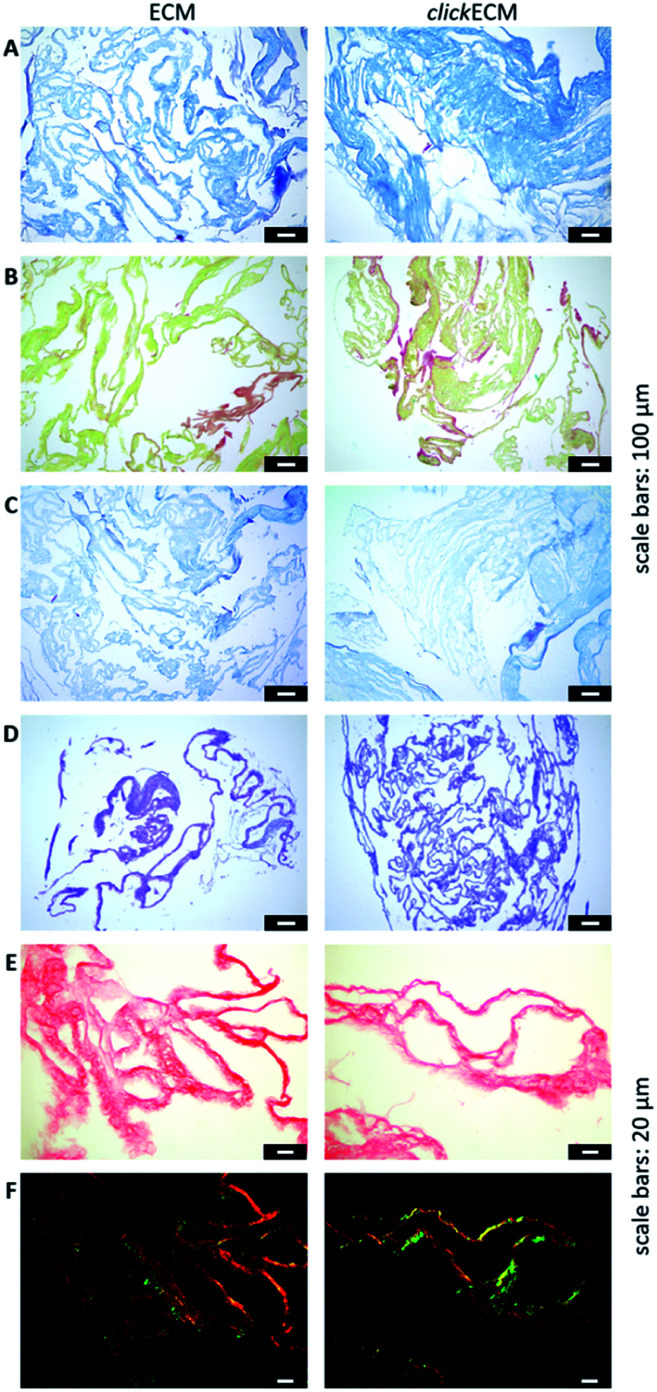
Representative light- and polarisation microscopic images from *n* = 3 of the histological evaluation of (click)ECM composition *via* multichrome stainings and stainings of individual ECM-specific components. (A) Masson–Goldner trichrome staining with Aniline Blue (collagens: blue), (B) modified Movat pentachrome staining (collagens: orange-yellow), (C) Ladewig staining (collagens: purple/violet, glycoconjugates: blue), (D) Alcian blue-periodic acid-Schiff (PAS) staining (glycoproteins and proteoglycans: purple), (E) Picro Sirius Red staining illuminated with bright field (collagens: red) and (F) Picro Sirius Red staining illuminated with polarised light (collagens: green/blue or red/yellow). Scale bars (A–D): 100 μm; (E and F): 20 μm.

In this study, histochemical multichrome stainings (namely Masson–Goldner trichrome staining with Aniline Blue (Fig. 2A), a modified Movat pentachrome staining (Fig. 2B) as well as Ladewig staining (Fig. 2C)) were used to show potential differences in the biochemical features of azide-modified clickECM.

The overall structure and coloration from those results was found to be similar for both ECM types regardless of the modification of the matrix with azides *via* MGE (Fig. 2A–C). This is in accordance with the overall conclusion of SDS-PAGE banding patterns and suggests that the expression of typical ECM biomolecules is not adversely affected. This is also in accordance with the findings of Ruff *et al.*^10^ who used a modified Movat staining on fibroblast-derived (click)ECM isolated after 21 days of cell culture. From previous studies, it is known that the monosaccharide Ac_4_GalNAz, which was also used in this study, is metabolically incorporated into *O*-linked glycoconjugates.^10–12,17^ Therefore, and in addition to the analysed multichrome stainings, an Alcian blue-periodic acid-Schiff (PAS) staining was performed to investigate the distribution and presence of glycoproteins and proteoglycans within (click)ECM thin sections. Fig. 2D revealed the homogeneous distribution of glycoconjugates over the entire structure equally for both unmodified ECM and azide-modified clickECM. The homogenous distribution of glycans was thus found to be consistent with the distribution of azides within clickECM structures (ESI Fig. 1†). This result complies with the findings of Ruff *et al.*^10^ who used a conventional Alcian blue staining on fibroblast-derived (click)ECM isolated after 21 days of cell culture.

Using Picro Sirius Red staining as an additional collagen-specific dye, we saw that collagens were also evenly distributed over both unmodified ECM as well as azide-modified clickECM (Fig. 2E). There was no obvious difference in the expression of this structural protein. Under polarised light, a double refraction was detected for both matrices (Fig. 2E). This effect was previously also observed by Schenke-Layland *et al.*^38^ who generated fibroblast-derived sheets on silicon-based nanostructures over a period of four weeks. Interestingly, the occurrence of the birefringence patterns observed in this current study suggest that even after seven days of static *in vitro* cell culture fibrillary collagens were deposited and that MGE apparently did not decelerate this process. However, the proportion of the detected colour hues in some images of the unmodified ECM seem to be different (greater proportion of red shades) from those detected by the modified clickECM (more green shades). There are studies published where researchers debate if the colour hues in the birefringence patterns can be used to distinguish between collagen type I (yellow/red) and collagen type III (green). Montes and Junqueira^39^ as well as Junqueira *et al.*^23^ stated that this is possible, while Lattouf *et al.*^40^ published results that suggest that the colouration strictly depends on the orientation of the collagen bundles. To investigate whether the methods allow differentiation between the two collagen types colours in (click)ECM or if the colours change when the sample orientation is altered, we acquired images of the identical areas with the same microscopic conditions before and after 90° stage rotation and compared the colouration (ESI Fig. 2†). It turned out that we observed the same effect as Lattouf *et al.* meaning that the red/yellow colour inverted into green and *vice versa* when the samples were rotated by 90°. Hence, we decided to use the Picro Sirius Red staining method solely for the detection of collagens in general and performed an immunohistochemical staining to study the appearance of the major ECM-specific collagen types.

**Fig. 3:**
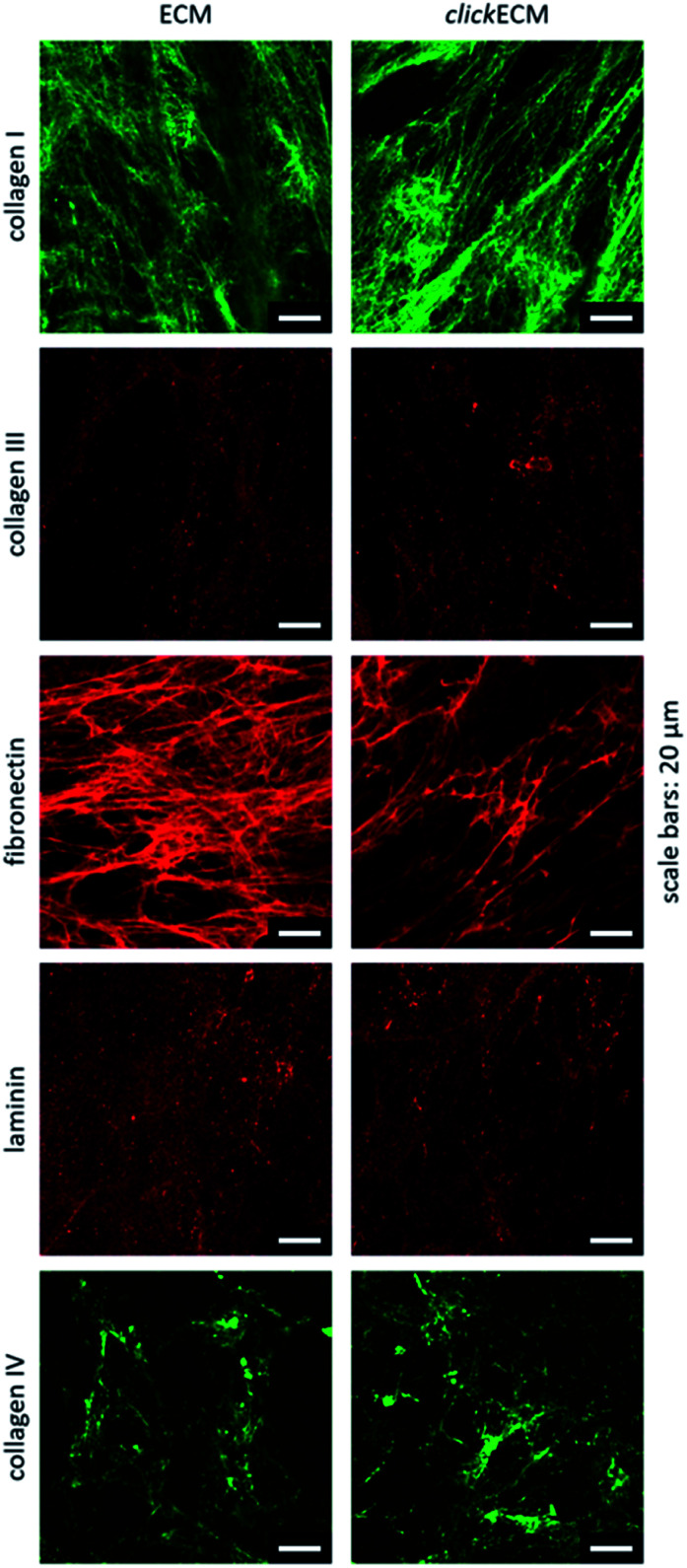
Representative images from *n* = 3 for the immunohistochemical analysis of the ECM-specific proteins collagen type I, collagen type III, collagen type IV, fibronectin, and laminin. All biomolecules were detectable in both unmodified ECM as well as azide-modified clickECM. Scale bars: 20 μm.

By staining for collagen type I, type III, and type IV as well as fibronectin and laminin as prominent candidates present in fibroblast-derived ECM, we were able to investigate the appearance of these biomolecules in both unmodified ECM as well as azide-modified clickECM. It turned out that all proteins were detectable regardless of the azide modification *via* MGE and the labelled proteins were evenly distributed over the entire structure of the stained (click)ECM samples (Fig. 3). These results are in good accordance with the findings of Ruff *et al.*^10^ and Keller *et al.*^17^ who also stained human fibroblast-derived (click)ECM for collagen type I and type IV as well as fibronectin.

The qualitative analysis methods tested in this study all proved to be of value in order to get an appreciation for the overall biomolecular nature of (click)ECM samples. The results gathered so far suggest that beyond the modification with azide groups MGE did not alter the biomolecule components present in clickECM compared to unmodified ECM.

### 3.2. Quantitative assessment of clickECM composition

After approaching the overall biochemical composition and distribution of the main ECM-specific biomolecules within (click)ECM, we attempted to identify reliable and useful methods to quantitatively assess the composition and determine the contents of the main (click)ECM components (proteins, soluble and insoluble collagens, sulphated glycosaminoglycans (sGAGs)). Natural ECM is characterised by different concentrations of various biomolecules, which in terms of their biochemical/biomolecular composition and constitution are quite diverse.^1,4,32,33,41^ This well-known characteristic of natural ECM was also seen in the SDS-PAGE banding pattern discussed above for cell-derived ECM (Fig. 1).

#### 3.2.1. Quantification of the total protein content within (click)ECM

As a starting point for quantitative assessment of the (click)ECM composition, we chose to begin with the quantification of the total protein content within (click)ECM. Thus, we consulted the literature and found that the majority of published studies on ECM determined the protein content using colorimetric assays such as the bicinchoninic acid (BCA) assay or the Bradford assay.^20,28,42–47^ These biochemical assays determine the total protein content in a solution through the interaction of the used dyes with the sample proteins leading to a photometrically measurable colour change of the sample solution. By comparing the measured absorbance of the protein sample to that of a standard protein of known concentration, the total protein content can be calculated.

**Fig. 4:**
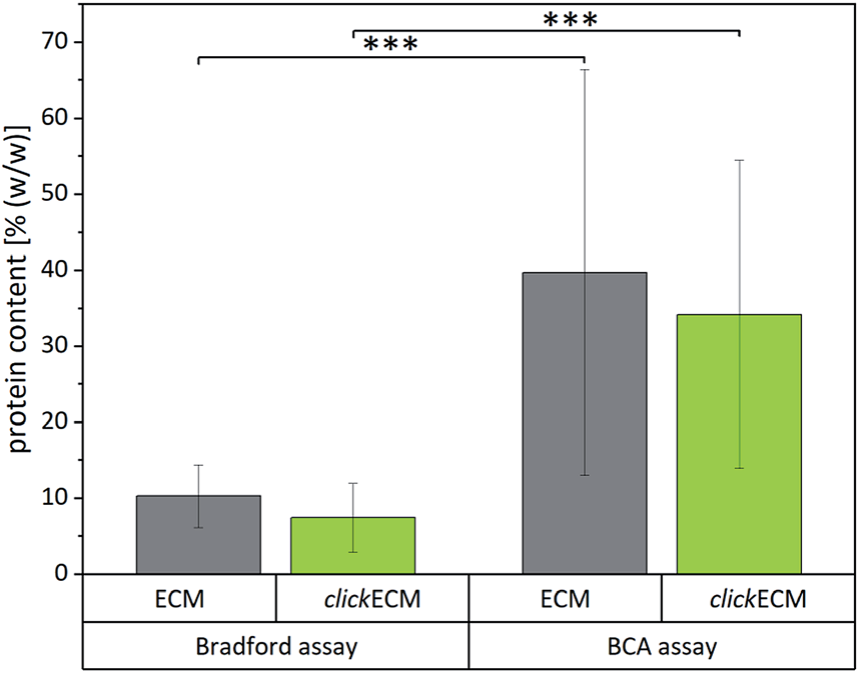
Quantification of the total protein content of unmodified ECM and azide-modified clickECM of three individual donors using the Bradford assay and the bicinchoninic acid (BCA) assay (*n* = 3). Results obtained from the two individual assays were statistically significant.

To investigate if colorimetric assays are suitable for the (click)ECM samples studied in this contribution, we chose two of the most frequently used assays (BCA and Bradford) and quantified the total protein content of (click)ECM samples derived from three individual donors in three independent measurements for each assay (Fig. 4). The colorimetric response in the Bradford assay is caused by the ability of the Coomassie Blue dye to bind protein causing a photometrically measurable colour shift while the BCA assay is based on the reduction of copper ions by the peptide bonds in the protein sample. For the latter, the formed Cu^+^ ions chelate with two molecules of BCA to form a coloured product, which can be photometrically measured.

Even though the results within the particular assays for the matrices derived from three individual donors were not statistically different, the results between the two assays varied significantly. Using the Bradford assay, the mean total protein content was found to be 10.3 ± 4.1% (w/w) for unmodified ECM and 7.5 ± 4.5% (w/w) for azide-modified clickECM while according to the BCA assay the samples contained 39.7 ± 26.7% (w/w) and 34.2 ± 20.3% (w/w) proteins in the case of unmodified ECM and azide-modified clickECM, respectively.

To evaluate if the determined values are representative, we compared them to the total protein content of human connective tissue published by Smith *et al.* who found the protein content to be 88% (w/w).^48^ As fibroblasts are the main cell type resident in connective tissue and mainly responsible for ECM secretion, the value published by Smith *et al.* seemed to be suitable as an approximate reference. Compared to this value, both assays would grossly underestimate the total protein content. We expected to see a protein content of fibroblast-derived (click)ECM to be somewhat smaller as it is known that some ECM precursor proteins are formed *in vitro* in soluble form but that they are not covalently integrated into the matrix network as mature fibres, *e.g.* in case of elastin, due to a lack of mechanical stimuli.^49,50^ A similar effect was reported for collagen, as the enzymatic conversion of procollagen to collagen does also not readily occur. While some insoluble collagen is assembled at the cell layer during *in vitro* cell culture, procollagen molecules secreted in the media may be lost when the culture medium is exchanged or the precursor molecules get eluted from the matrix due to the washing steps during (click)ECM isolation.^45^

However, the big differences between the protein content of connective tissue reported by Smith *et al.* and the results obtained from the two colorimetric assays performed in this study make the assays very likely not trustworthy for the analysis of fibroblast-derived (click)ECM studied in this work. This assumption is furthermore strengthened by the rather large standard variations.

We presume that there are three main reasons for the large variations obtained with the two commercially available assays which all originate from the insoluble, highly complex assembled nature of (click)ECM samples. First, in order to enable the underlying chemical reaction of the respective assay and furthermore to keep the optical light path clear for the photometrical detection of the sample absorbance, it is essential that the samples are completely dissolved. Previously, it was shown that the homogenisation protocol used in this study results in (click)ECM fragment sizes in the range of 7.62 to 31.29 μm.^17^ Due to the fact that these (click)ECM fragments are insoluble, it was not expected that they are dissolved in the course of the two colorimetric assays investigated. To confirm this, light microscopic analysis was done on the reaction solutions and the acquired images (ESI Fig. 3†) revealed that there were still insoluble (click)ECM fragments present which very likely contributed to the varying results.

In addition to the differences in mechanism between the assays, the highly complex protein composition may also be a reason for significant variations between the assays. In their review, Sapan *et al.*^51^ described several chromogenic protein assays and emphasised that the results of these assays are often markedly influenced by the protein-to-protein variation of the sample, which could also be a reason for the huge variations between the BCA and the Bradford assay used in this study.

The third reason we suspect, which probably significantly influences the outcome of the chromogenic protein assays, arises from the used standard proteins. Both commercially purchased assays used in this study use the small soluble bovine serum albumin (BSA) as standard. It is known that if the protein used as a standard does not react to the assay dye in a very similar way, the concentration measured will be inaccurate.^51^ A matching standard for ECM analysis would – in our sole opinion – have to mimic the tissue and donor specific complex protein composition very closely in order to provide reliable results. As it is neither possible to fully dissolve the sample nor to match the standard with the complex sample protein composition, we concluded that the assays are not suited for the cell-derived (click)ECM studied in this work.

To circumvent the solubility and complexity issue, we were looking for an alternative method to quantify the total protein content within the (click)ECM investigated in this study where neither the insolubility nor the lack of a representative standard matters. Such a method, which has been almost universally used for many years in the fields of basic and applied food and nutrition sciences, is the determination of the total protein content based on the total nitrogen content.^52–54^ For this determination, the amount of total nitrogen in the sample is quantified by the Kjeldahl or a comparable method and then multiplied with a “nitrogen-to-protein conversion factor”. The most commonly cited conversion factor is 6.25 which dates back to a study published by Mulder in 1839.^55^ In this study, Mulder suggested a common elementary composition of protein (C_40_H_62_N_10_O_12_) and found the average nitrogen content of proteins to be about 16%. The reciprocal value of this nitrogen content finally resulted in the generic conversion factor of 6.25 that has been used ever since.^53,55^ Several years later, Jones postulated that the use of 6.25 as a single factor is misleading due to the fact that not all nitrogen in biological samples is found in proteins and that the nitrogen content of specific amino acids varies depending on the molecular weight of the amino acid and the number of nitrogen atoms in it.^54^ He therefore suggested specimen-specific “Jones factors” for the most commonly eaten foods ranging from 5.18 to 6.38. For gelatin (the denatured derivative of collagen^56–59^), he recommended 5.55 as suitable conversion factor.^53,54^ As collagen was found to account for one of the major ECM-specific components in (click)ECM (Fig. 1 and 2),^1,32,33^ we contemplated this factor as a potential suitable match and compared it to the theoretical nitrogen-to-protein conversion factors of collagen (type I, III, and IV), fibronectin, and laminin. The factors for the three collagen types as well as for fibronectin and laminin were calculated based on the amino acid sequences of the mature ECM proteins derived from the universal protein database (UniProt^60^) (ESI Part 3.1.2: Calculation of nitrogen-to-protein conversion factors based on ECM-specific proteins†). Furthermore and due to the fact that fibroblasts are the main cell type found in the dermis of skin, we also consulted literature for contributions where the amino acid composition of human skin was analysed and converted the published values in the same way as for the individual ECM-specific biomolecules derived from UniProt.^60^ We chose the work of Bornstein and Piez^61^ who analysed human infant skin collagen as well as the study of Miyahara *et al.*^62^ who investigated the amino acid composition of purified gelatin from human female skin (age 0). The full list of reviewed publications is shown in the ESI (Table S2).† All of the obtained factors for individual ECM-specific biomolecules as well as complex tissues are listed in Table 1.

**Table 1:** Protein-specific nitrogen-to-protein conversion factors calculated from either the mature amino acid composition of the five ECM-specific proteins collagen (COL) type I, III, IV, fibronectin (FN) and laminin (LN) or from the amino acid sequences found in literature on human skin

Jones factor	COL			FN	LN	Human infant skin	
	I	III	IV			Bornstein and Piez^61^	Miyahara *et al.*^62^
5.55	5.25	5.31	5.69	5.88	5.66	5.45	5.65

The calculated factors spanned a range from 5.25 (collagen type I) to 5.88 (fibronectin), meaning that the mentioned Jones factor for gelatin (5.55) as well as both factors calculated for human skin collagens (5.45 and 5.65) lie within this range. We then quantified the nitrogen content of the (click)ECM generated from three individual donors by catalytic combustion of the samples followed by the separation and analysis of the resulting combustion gases using a thermal conductivity detector (TCD). Next, we converted the measured nitrogen contents into total protein contents using the Jones factor for gelatin as well as the calculated theoretical factors for the ECM-specific biomolecules (collagen types I, III, IV, fibronectin, and laminin) and the factors for human skin gelatin listed in Table 1. The results are depicted in Table 2.

**Table 2:** Total protein contents [% (w/w)] (*n* = 3) derived from the conversion of the measured nitrogen content by the specific nitrogen-to-protein conversion factors for gelatin (derived from Jones (JF = Jones factor)), collagen (COL) type I, III, IV, fibronectin (FN), and laminin (LN) (derived from UniProt^60^) as well as for human skin collagen (derived from Bornstein and Piez^61^ as well as Miyahara *et al.*^62^)

	Jones factor	COL			FN	LN	Human infant skin	
		I	III	IV			Bornstein and Piez^61^	Miyahara *et al.*^62^
ECM	56 ± 4	53 ± 4	54 ± 4	57 ± 4	59 ± 4	57 ± 4	55 ± 4	57 ± 4
clickECM	58 ± 4	54 ± 4	55 ± 4	59 ± 4	61 ± 4	59 ± 4	57 ± 4	59 ± 4

Derived from the total protein contents listed in Table 2, the mean protein content can be expected to lie within the range of 53 ± 4% (w/w) and 59 ± 4% (w/w) for unmodified ECM and in the range of 54 ± 4% (w/w) and 61 ± 4% (w/w) for azide-modified clickECM. There was no statistically significant difference found between the unmodified ECM and the azide-modified clickECM. Statistical analysis of the resulting protein content obtained from the individual conversion factors can be found in the ESI (Table S3).† This analysis confirms the expectation that there will be statistically significant differences between the results depending on the used conversion factors.

Deciding which conversion factor is the most appropriate one is not trivial as ECM is known to be a highly complex and dynamic mixture of biomolecules composed of up to 300 different proteins in tissue-specific combinations and concentrations.^30^ Since the exact composition of the (click)ECM investigated in this study is not itemised in such detail, it is possibly not feasible to choose one specific factor. In fact, we suggest considering a tissue-specific range. In spite of this limitation and based on the small standard deviations of the nitrogen quantification, the obtained values in this study still appear significantly more robust and reliable than the results derived from the two colorimetric assays. For this reason, we are convinced that this method is, despite of the limitations arising from the uncertainty in terms of the exact ECM protein composition and the amount of non-protein nitrogen present in the sample, still better suited to quantify the total protein content of the (click)ECM investigated in this study than the tested colorimetric assays.

According to our analysis, the protein contents of the *in vitro* generated fibroblast-derived (click)ECM generated in this study were slightly lower than the protein content of natural connective tissue published by Smith *et al.*^48^ (88% (w/w)). As mentioned before, this lower protein content was expected and lies within a realistic range.

In general, another method for the direct quantification of the protein content within biological samples is the analysis and quantification of amino acids. For this method, samples are first hydrolysed with hydrochloric acid and the amino acids are then separated by ion chromatography. Next, the separated amino acids are converted with ninhydrin in a post-column derivatisation and the resulting amino acid derivatives can then be detected using a photometric detector. Quantification of the amino acid content and hence of the protein content is finally completed using amino acid standards.^63^ To apply this method as an additional technique to analyse the total protein content of (click)ECM would be desirable. However, until now, the manual generation, isolation, and processing of clickECM is very labour-intensive and yields a very limited amount of sample material. Therefore, it was not possible in this study to apply both methods. However, we overall conclude that the approach of quantifying the total protein content of (click)ECM *via* elementary analysis and conversion of the nitrogen content into the total protein content seems to be a robust, reliable, and practical method where neither the insolubility of complex biological matrices nor the lack of a representative standard is a challenge.

#### 3.2.2. Quantification of the collagen content within (click)ECM *via* the hydroxyproline (HP) content

For the next step in the quantitative assessment of (click)ECM composition, we chose to quantify the total collagen content. Collagen is one of the main classes of structural fibre proteins in connective tissues in vertebrates and therefore also in fibroblast-derived ECM.^24,48,64–66^ The family of collagens contains a variety of different collagen subtypes where type I and type III for instance belong to the group of fibrillary collagens mainly found in connective tissue and bones, while type IV belongs to the basement membrane group.^1,24^

The primary structure of this large family of glycoproteins is characterised by a repeating tripeptide amino acid sequence glycin–X–Y, where X is frequently proline and Y often hydroxyproline (HP). During biosynthesis, a triple helix of three procollagen α-chains is formed. This soluble precursor procollagen is secreted into the extracellular space where it is enzymatically converted into tropocollagen. During fibrillogenesis, collagen fibrils are formed *via* the covalent cross-linking of several tropocollagen molecules. These molecules eventually form collagen fibres, when multiple collagen fibrils congregate.^1^

The α-amino acid HP occurs almost exclusively in collagen and only in very little amounts in elastin. Due to the abundance of collagen in most mammalian tissues and the fact that elastins are not covalently integrated into cell-derived ECM as mature fibres under standard *in vitro* cell culture conditions due to a lack of mechanical stimuli,^49,50^ the protein-bound HP content can therefore be used for collagen quantification with an acceptable level of accuracy.^24,67–71^

To quantify the collagen content, samples are predominantly hydrolysed with hydrochloric acid, whereby polypeptides are broken down into individual amino acids, which are then oxidised with chloramine T. The oxidation product forms a red condensation product with *p*-dimethylaminobenzaldehyde, which can be quantified by photometric absorption measurement at 555 nm.^24^ The measured HP content can then be converted into the collagen content by dividing the measured HP by a specific conversion factor (HP [% (w/w)]/100) similar to the above described procedure for quantifying the total protein content based on the conversion of nitrogen into protein.

In the SDS-PAGE banding patterns in Fig. 1 as well as in the (immune)histochemical stainings in Fig. 2 and 3 it can be appreciated that the biomolecular composition of (click)ECM is fairly complex. To find a suitable HP-to-collagen conversion factor for the estimation of the collagen content in samples with such complex compositions, we first consulted the literature for studies in which the HP content of complex samples like skin was reported. The complete list can be found in the ESI (Part 3.1.3: Overview over reviewed literature for the calculation of specific nitrogen-to-protein and hydroxyproline-to-collagen conversion, Table S2).†

It turned out that there was not one universally applicable conversion factor to find but several slightly different factors, which also varied depending on the species where the collagen was extracted. For example, Capella-Monsonis *et al.*^24^ suggested 0.135 as conversion factor, as they claim that this is the percentage (13.5% (w/w)) of HP in collagen type I in mammalian tissues. They also pointed out that for fish tissues or other collagen types, the appropriate hydroxyproline content should be used. This species dependency was also reported by Hofman *et al.*^71^ who furthermore rated mammalian collagens to contain 14% HP. Neuman and Logan^70^ also collected HP contents from the literature which they listed as “best values in literature of gelatin” which spanned a range of 12.9–14.6% and found their own values for gelatins and collagens (13–14%) to lie in this range. A similar range (10–14%) was reported for collagen extracted from skin by Edwards and O'Brien Jr^67^ who assumed an average amount of 12.5 g HP/100 g protein for collagen.

Another important contribution was made by Etherington and Sims^72^ who showed that the individual collagen types in meat and meat products contain quite different HP contents. Amongst others, they listed HP contents for type I (13.1%), type III (17.4%), and type IV (16.6%). They also pointed out that due to the fact that all chemical determinations are made on the free amino acids after hydrolysis of the protein, HP contents should be corrected for the (formal) addition of one water molecule to each amino acid residue so that the values represent the free amino acids. The value obtained should therefore be understood as mass of hydrolysed hydroxyproline generated per mass of unhydrolysed protein rather than as a hydroxyproline content.

Since the exact collagen composition and contents of the individual collagen types within the (click)ECM studied in this current work remain unknown as of yet, we approached the collagen contents by choosing conversion factors from the literature, which seemed to be suitable for a human fibroblast-derived ECM. Since fibroblasts are the main cell type found in the dermis of skin, we used the HP content from the amino acid analysis of human infant skin collagen published by Bornstein and Piez^61^ (13.5%) as well as the HP content of human infant skin (13.3%) published by Miyahara *et al.*^62^ as complex collagen assemblies. As collagen comprises 70–80% of the dry weight of human skin dermis whereby collagen type I and type III are the most abundant types,^73^ we also adduced the HP content from the amino acid analysis of human collagen type I (0.135) published by Capella-Monsonis *et al.*^24^ and mammal collagen type III (0.180) derived from the work of Chung and Miller.^74^ The total collagen contents derived from the calculated HP-to-collagen conversion factors are listed in Table 3.

**Table 3:** Collagen contents [% (w/w)] (*n* = 2) derived from the conversion of the measured hydroxyproline (HP) content by the specific HP-to-collagen conversion factors for collagen (COL) type I, III, (derived from Capella-Monsonis *et al.*^24^ and Chung and Miller^74^) as well as for human infant skin collagen (derived from Bornstein and Piez^61^ as well as Miyahara *et al.*^62^)

		COL		Human infant skin collagen	
		I	III		
Reference		Capella-Monsonis *et al.*^24^	Chung and Miller^74^	Bornstein and Piez^61^	Miyahara *et al.*^62^
HP-to-collagen conversion factor		0.135	0.180	0.135	0.133
Collagen content [% (w/w)]	ECM	16.5 ± 3.4	12.4 ± 2.5	16.5 ± 3.4	17.9 ± 3.4
	clickECM	24.9 ± 5.4	18.7 ± 4.0	24.9 ± 5.4	25.2 ± 5.5

Derived from the collagen contents listed in Table 3, the mean collagen content can be expected to lie within the range of 12.4 ± 2.5% (w/w) and 17.9 ± 3.4% (w/w) for unmodified ECM and in the range of 18.7 ± 4.0% (w/w) and 25.2 ± 5.5% (w/w) for azide-modified clickECM. Statistical analysis indicated that there was no statistically significant difference between the results derived from the conversion factor calculated based on the data published by Bornstein and Piez^61^ (0.135) and the conversion factor calculated from the work of Miyahara *et al.*^62^ (0.133). Furthermore, statistical analysis of the collagen quantification results indicated that azide-modified clickECM contained significantly more collagen than the unmodified ECM. So far, there is – to the best of our knowledge – no data on this effect of MGE with Ac_4_GalNAz published. Hence, the exact reason for this remains unknown at this moment. Except for the added monosaccharides (unmodified ECM: GalNAc (C_8_H_15_NO_6_) and azide-modified clickECM: Ac_4_GalNAz (C_16_H_22_N_4_O_10_)), cells were treated the exact same way during *in vitro* cell culture.

Deciding which conversion factor is the most appropriate one is not trivial as ECM is known to be a highly complex and dynamic mixture of collagens.^73^ Since the exact composition of collagens within the (click)ECM investigated in this study is not itemised in such detail, it is very likely not feasible to choose one specific factor. In fact, we suggest to consider a feasible tissue-specific range, similar to the reasoning described for the nitrogen-to-protein conversion factor.

To investigate whether or not there are also soluble collagens present in the *in vitro* generated (click)ECM, we performed the Sircol™ Soluble Collagen Assay. This assay assesses newly synthesised collagen, which is not cross-linked yet. It turned out that no soluble collagen was found neither within unmodified ECM nor in azide-modified clickECM (*n* = 3). The reason for this could be that soluble collagens were either already dissolved out of the matrix network during *in vitro* cell culture by the cell culture medium^45^ or that they got washed out by the excessive washing steps during decellularisation. The observed compliance between the (click)ECM band patterns and the collagen band patterns in stained protein gels (Fig. 1) suggests that collagens make up the majority of the matrix protein. With the results gained from the Sircol™ assay it seems like the collagen within (click)ECM isolated after only seven days of static cell culture is already cross-linked and can therefore not be dissolved by neutral buffers and acids. This assumption is also in accordance with the results obtained from the Picro Sirius Red staining under polarised light (Fig. 2E, F and S2†). The occurrence of the birefringence patterns already suggested that even after seven days of static *in vitro* cell culture fibrillary collagens were deposited.

Smith *et al.* quantified the amounts of collagen in dermal connective tissue of children (age < 1 year). In that study, the total collagen content of 72.3% (w/w) consisted of 8.6% (w/w) soluble and 63.7% (w/w) insoluble collagen. Given the fact that the (click)ECM investigated in this current study was isolated after seven days already, we expected the collagen content to be significantly lower than the values published by Smith *et al.* for dermal connective tissue of infants. As it is known that the total collagen content increases with age,^48^ the values observed in this current study seem to lie within a realistic range.

In general, alternative methods based on a different reaction mechanism would be of value to validate the results obtained in this study. Quantitative ELISA of the individual collagen types might be conceivable. However, all known types of collagen would have to be tested which would however be very expensive. Nuclear magnetic resonance spectroscopy (NMR) for instance could also be a suitable yet also quite expensive method^72^ for the analysis and quantification of amino acids.

#### 3.2.3. Quantification of the glycosaminoglycan content within (click)ECM

Polysaccharides in the form of glycans are an important class of ECM biomolecules.^1,4^ Qualitative assessment of the glycan distribution shown in Fig. 2D indicated that GAGs are homogeneously distributed over the entire (click)ECM. Quantitative assessment of the sulphated glycosaminoglycan (sGAG) content was carried out using the Blyscan™ assay (*n* = 3). The sGAG levels of the *in vitro* generated (click)ECM are shown in Table 4. sGAG content of unmodified ECM was found to be 3.1 ± 0.6% (w/w) while the sGAGs content in azide-modified clickECM was determined to be 3.3 ± 0.9% (w/w). There was no statistically significant difference found between these results, which give rise to the assumption that sGAG expression is not impaired by MGE.

**Table 4:** Characterisation results for unmodified ECM and azide-modified clickECM. The total protein content was analysed *via* elementary analysis and nitrogen-to-protein conversion. The total collagen content was assessed *via* the amount of hydroxyproline (HP) and sulphated glycosaminoglycan (sGAG) content was determined using the Blyscan™ assay. Soluble collagens were not detected with the Sircol™ Soluble Collagen Assay. Thus, the results are not listed

	Total protein content [% (w/w)]	Total collagen content [% (w/w)]	Total sGAG content [% (w/w)]
ECM	53 ± 4 to 59 ± 4	12.4 ± 2.5 to 17.9 ± 3.4	3.1 ± 0.6
clickECM	54 ± 4 to 61 ± 4	18.7 ± 4.0 to 25.2 ± 5.5	3.3 ± 0.9
Statistical difference	n.s.	***	n.s.

The used Blyscan™ assay is a commercially distributed version of the 1,9-dimethyl-methylene blue (DMMB) assay, which detects sGAG based on the phenomenon of metachromasia, with the characteristic blue of the cationic DMMB dye shifting to a violet hue when the dye binds to polyanionic substrates such as sGAG.^75,76^ It is known that artifacts in sGAG measurements have the potential to substantially affect results and interpretations due to the presence of cell- and matrix-associated polyanionic contaminants. Zheng and Levenston^75^ postulate that this could overestimate the actual sGAG contents, particularly at early time points in culture or for samples with a relatively little sGAG content. To circumvent such a risk of false positive results for the (click)ECM isolated after seven days of *in vitro* cell culture studied in this work, we determined the pH of the used Blyscan™ dye reagent and found it to be 1.6. Hence, it was close to the pH of 1.5 recommended by Zheng and Levenston^75^ and sufficiently low to guarantee quantitative protonation of carboxylic acid groups present *e.g.* in ECM proteins. We therefore assume that the risk of an overestimation of the sGAG content is very low and hence the results from this current study seem to be accurate. Additionally, compared to the study published by Schenke-Layland *et al.*,^38^ who investigated the sGAG content of fibroblast-derived ECM sheets generated on silicon-based nanostructures over a period of 4 weeks to be 2.4% (w/w), the results obtained in this current study were found to be in the same order of magnitude.

As mentioned above, the Blyscan™ assay is the most frequently used method to quantify sGAG contents. Other, non-colorimetric methods for sGAG quantification such as liquid chromatography-tandem mass spectrometry techniques would be a valuable tool to verify the determined contents, however transferring insoluble (click)ECM into sample solutions compatible with these methods bears a high risk of losing GAG molecules due to the series of extraction and recovery steps necessary.

## 4. Conclusion

In this contribution, we investigated and evaluated widely used analytical methods for the analysis of the biomolecular composition of chemically modified cell-derived matrices obtained by MGE. For this purpose, (click)ECM was analysed in a qualitative manner using SDS-PAGE and a number of (immune)histochemical methods in combination with light microscopy, polarisation microscopy, and fluorescence microscopy to study the appearance and distribution of the main ECM-specific biomolecules. Subsequently, we quantified the contents of total protein, total and soluble collagen, and sGAGs. The results are combined in Table 4.

As listed in Table 5, we re-assessed several frequently used qualitative and quantitative methods to identify reliable analytical methods for the analysis and characterisation of the biomolecular composition of (click)ECM obtained by MGE.

**Table 5:** Overview over all tested methods and evaluation of their applicability to characterise fibroblast-derived (click)ECM. A plus sign in the applicability column represents in our opinion a method well-suited for the analysis of fibroblast-derived (click)ECM. Methods labelled with a minus sign on the other hand should not be used in our sole opinion. Superscripted letters indicate additional recommendations or limitations and the respective explanations are listed below

	Method	Aim	Applicability
Qualitative	SDS-PAGE	Banding pattern	+^a^
	Histochemical staining methods in combination with light, polarisation and fluorescence microscopy	ECM-specific biomolecules	+
	Immunohistochemical stainings	ECM-specific biomolecules	+
Quantitative	Bradford protein assay	Proteins	−
	BCA protein assay	Proteins	−
	Elementary analysis of N content + conversion into protein content	Proteins	+^b^
	Collagen content *via* hydroxyproline quantification	Insoluble (cross-linked) collagens	+^c^
	Soluble collagen content *via* Sircol™ assay	Soluble (freshly synthesised) collagens	+^c^^,^^d^
	Sulphated glycosaminoglycan content *via* Blyscan™ assay	sGAGs	+^c^

a Only components that can penetrate the gel can be found. Consequently, sample preparation is crucial.

b The method in general appears to be well suited, yet we suggest to consider the range of ECM-specific nitrogen-to-protein conversion factors instead of a single conversion factor.

c The method in general appears to be well suited, yet it would be desirable to apply an alternative method for the verification of the results.

d This method in general appears to be applicable, however (click)ECM produced with the above stated protocol seems to contain no soluble collagens.

All of the investigated qualitative techniques proved to be of value in order to get an appreciation for the overall biochemical nature of (click)ECM samples. In our opinion, they can be considered as a valuable tool to study the overall biomolecular composition and architecture of the analysed cell-derived matrices. However, these methods are not suited for the detection of smaller differences between unmodified ECM and azide-modified clickECM.

The quantitative analytical methods applied allowed to measure the protein, collagen, and sGAG contents as they make up the main ECM compounds as indicated by the qualitative methods. In case of protein quantification we had to implement a method based on the conversion of the measured nitrogen content into the total protein content because the two tested chromogenic protein assays (Bradford and BCA) turned out to be unsuitable for the analysis of highly complex and insoluble cell-derived (click)ECM studied in this work. All other tested methods (quantification of the soluble collagen content as well as the sGAG content) proofed to be applicable.

In summary, we saw that MGE, beyond the modification of the matrix with azide groups, increased the amount of collagen within clickECM compared to the unmodified ECM. Between all other analysed parameters, no statistical difference were detected in direct comparison to the unmodified ECM.

Ultimately, our results show that not all analytical methods routinely used in the literature are readily suitable for the analysis of insoluble and complex composed ECMs and CDMs. Instead, it has been shown that it is essential to critically assess the suitability of a particular assay in order to reliably investigate the respective feature. The same is true for the selection of the appropriate conversion factors.

## Conflicts of interest

There are no conflicts to declare.

## Supplementary Material

RA-010-D0RA06819E-s001
