# Supplementary material for: Eclectic characterisation of chemically modified cell-derived matrices obtained by metabolic glycoengineering and re-assessment of commonly used methods
Source: RSC Adv. 2020 Sep 23;10(58):35273–86. doi: 10.1039/d0ra06819e (PMC9056897; doi:10.1039/d0ra06819e)
Supplement: RA-010-D0RA06819E-s001 [file RA-010-D0RA06819E-s001.pdf]

Electronic Supplementary Material (ESI) for RSC Advances.
This journal is © The Royal Society of Chemistry 2020
Please do not adjust margins

Received 00th January 20xx,
Accepted 00th January 20xx

DOI: 10.1039/x0xx00000x

Electronic Supplementary Information (ESI)

Eclectic Characterisation of Chemically Modified Cell-Derived Matrices Obtained by Metabolic Glycoengineering and Re-Assessment of Commonly Used Methods

Silke Keller[a,b], Anke Liedek[a], Dalia Shendi[c], Petra J. Kluger[d], Monika Bach[a†], Günter E. M. Tovar[a,b,*], Alexander Southan[a,*]

[a.] *Institute of Interfacial Process Engineering and Plasma Technology IGVP, University of Stuttgart, Nobelstraße 12, 70569 Stuttgart, Germany.*
[b.] *Fraunhofer Institute for Interfacial Engineering and Biotechnology IGB, Nobelstraße 12, 70569 Stuttgart, Germany*
[c.] *Department of Biomedical Engineering, Worcester Polytechnic Institute, Worcester, MA, USA*
[d.] *Reutlingen University, School of Applied Chemistry, Alteburgstraße 150, 72762 Reutlingen, Germany*
[*] Corresponding authors
[†] Present Addresses: University of Hohenheim, Core Facility, Module 3: Analytical Chemistry Unit, Emil-Wolff-Str. 12, 70599 Stuttgart, Germany.

Please do not adjust margins

Please do not adjust margins

ARTICLE

Table of Contents

1. Materials and Instruments 3

1.1. Materials 3

1.2. Instruments 3

2. Qualitative Assessment of *click*ECM Composition 4

2.1. Azide Modification of Fibroblast-Derived Extracellular Matrix via Metabolic Glycoengineering 4

2.2. Polarisation Microscopy 5

3. Quantitative Assessment of *click*ECM Composition 6

3.1. Quantification of the total Protein Content within (*click*)ECM 6

3.1.1. Quantitative Assessment of *click*ECM Composition 6

3.1.2. Calculation of Nitrogen-to-Protein Conversion Factors and Hydroxyproline-to-Collagen Conversion Factors Based on ECM-Specific Protein 7

3.1.3. Overview over reviewed literature for the calculation of specific nitrogen-to-protein conversion factors 8

3.1.4. Statistical Analysis of the Calculated Protein Concentrations Within (*click*)ECM 9

3.2. Quantification of the Collagen Content Within (click)ECM via the Hydroxyproline (HP) Concentration 9

4. Supporting Information References 10

Please do not adjust margins

Please do not adjust margins

1. Materials and Instruments

1.1. Materials

The following materials were obtained from the commercial sources given in parentheses: Cellstar® tissue culture flasks, Cellstar® cell culture multiwell plate, 12 and 96 wells, 50 mL-centrifugation tubes and ⌀ 145 mm-Cellstar® dishes (Greiner Bio One GmbH, Frickenhausen, Germany). Trypsin (supplemented with Versene (Ethylenediaminetetraacetic acid, EDTA)), aqueous mounting medium (Lab Vision™ PermaFluor™), fetal calf serum (FCS) (Gibco), 1 % penicillin/streptomycin (P/S), glass slides (Menzel glasses),Click-iT® Cell Reaction Buffer Kit, Alexa Fluor® 488-alkyne, the primary antibody rabbit anti-collagen III, the primary antibody rabbit anti-laminin, the secondary antibody goat anti-mouse-Alexa Fluor® 488, the secondary antibody goat anti-rabbit-Alexa Fluor® 647, Novex™ 8-16 % Tris-Glycine Mini Gels, WedgeWell™ format, 10-well, PageRuler™ Plus Prestained Protein Ladder, 10 to 250 kDa, Coomassie Brilliant Blue R-250 Imperial™ Protein Stain, Novex™ Tris-Glycine SDS Sample Buffer (2X), Novex™ Tris-Glycine SDS Running Buffer (10X), DWK Life Sciences Wheaton™ V Vial™ with solid-top screw PTFE caps, Pierce™ BCA protein assay kit and the Coomassie Plus-Assay-Kit (Thermo Fisher Scientific, Darmstadt, Germany). The primary antibody rabbit anti-fibronectin, the primary antibody mouse anti-collagen I, the primary antibody mouse anti-collagen IV (abcam, Cambridge, UK). Amicon® Ultra-15, PLGC Ultracel-PL Membran, 10 kDa (Merck, Darmstadt, Germany). N-azidoacetylgalactosamine-tetraacylated ($Ac_4GalNAz$), innuSPEED lysis tube type A (Analytik Jena, Jena, Germany), phosphate-buffered saline without calcium chloride and magnesium chloride (PBS^-), bovine serum albumin (BSA, dissolved in PBS^-), Triton X-100, Tween® 20, sodium dodecyl sulfate (SDS, 20 % in ultrapure water, H_2O), Na-L-ascorbate, hydrochloric acid (HCl), saponin, fluorescamine, 1-Ethyl-3-(3-dimethylaminopropyl)carbodiimide (EDC), hydrogen peroxide (~ 30 %), 2-(Nmorpholino)ethanesulfonic acid (MES-45 buffer), ammonia solution (~ 24 %), ethanol, isopropanol, N-Acetyl-D-galactosamine (GalNAc ~ 98 %), papain from papaya latex, buffered aqueous suspension, Direct Red 80, Picric acid solution (0.9 - 1.1 % (alkalimetric)) (Sigma Aldrich Chemie GmbH, Taufkirchen, Germany). The formalin solution (Roti®-Histofix), the clearing agent (RotiClear®), xylene, chloramine T trihydrate (≥98 %, p.a.), 4-(Dimethylamino)-benzaldehyde (≥98 %, p.a., ACS), citric acid monohydrate (≥99,5 %, Ph.Eur.), sodium acetate (≥99 %, p.a., ACS, anhydrous), perchloric acid (ROTIPURAN® 70 %, p.a.), tri-sodium citrate dihydrate (≥99 %, Ph.Eur.), L-4-hydroxyproline (≥99 %, for biochemistry), disposable syringe Injekt® With Luer-Lock fitting, 3 mL (Carl Roth, Karlsruhe, Germany). Dulbecco's Modified Eagle Medium (DMEM) (Biochrom AG, Berlin, Germany). Imaging dishes (ibidi µ-dishes) (ibidi GmbH, Planegg/Martinsried, Germany). Glass cover slips (R. Langenbrinck, Emmendingen, Germany). PTFE syringe filter (⌀ 0.2 µm) and the mounting media (Isomount) (VWR International GmbH, Bruchsal, Germany). Ultrapure water was withdrawn from a Barnstead GenPure xCAD water purification system (Thermo Fisher Scientific, Darmstadt, Germany). Masson-Goldner-trichrome, Ladewig, MOVAT pentachrome (original) and Alcian Blue-PAS staining kits (Morphisto, Frankfurt am Main, Germany). Sircol™ soluble collagen assay and Blyscan™ sulphated glycosaminoglycan assay (Biocolor, Carrickfergus, UK).

All commercial reagents were used as received without further purification.

Collagen type I was isolated from rat tails at the Fraunhofer Institute for Interfacial Engineering and Biotechnology IGB and provided with a concentration of 6 mg/mL.

1.2. Instruments

Confocal laser-scanning-microscope LSM 710 (Carl Zeiss AG, Oberkochen, Germany). Inverted microscopes ECLIPSE TS100 (Nikon, Düsseldorf, Germany). Polarisation microscope Leitz DM RXP (Leica, Wetzlar, Germany). Multifunctional microplate reader Infinite M200Pro (Tecan Group AG, Männedorf, Switzerland). Freeze dryer Alpha 2-4 LSCbasic (Martin Christ, Osterode am Harz, Germany). Bead mill SpeedMill PLUS (Analytik Jena, Jena, Germany), Balance XS 205, DualRange (Mettler Toledo, Columbus, OH USA). Orbital shaker Polymax 1040, 10° tilt angle (Heidolph, Schwabach, Germany). Mini-Cell Electrophoresis System XCell SureLock, the microtome HM340E and the centrifuge Heraeus Multifuge 3 S-R (Thermo Fisher Scientific, Darmstadt, Germany). Ultrasonic lab device UP200H (200W, 24kHz) and the ultrasonic sonotrode S3, titan, ⌀ 3 mm, length 100 mm (Hielscher Ultrasonics GmbH, Teltow, Germany).

This journal is © The Royal Society of Chemistry 2020

Please do not adjust margins

Please do not adjust margins

2. Qualitative Assessment of *click*ECM Composition

2.1. Azide Modification of Fibroblast-Derived Extracellular Matrix via Metabolic Glycoengineering

In a first step, the chemical accessibility, reactivity, and specifity of the reactive groups was invesitigated. For this purpose, (*click*)ECM sections were stained with the fluorophore and analysed using confocal laser scanning microscopy (cLSM) in combination with differential interference contrast (DIC) microscopy (**Fehler! Verweisquelle konnte nicht gefunden werden.**).

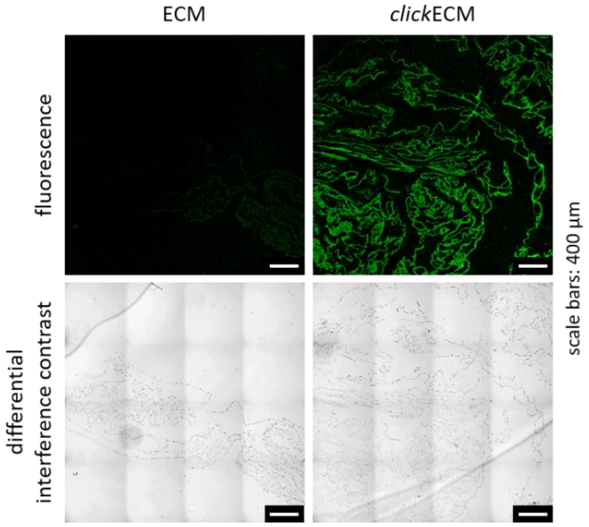

Figure S 1: Representative cLSM tilescan images of (*click*)ECM sections stained with an alkyne-modified fluorophore (Alexa Fluor® 488-alkyne). Azide groups (displayed in green in the upper row images), which were incorporated into the *click*ECM by metabolic glycoengineering (MGE), were detectable via copper-catalyzed 1,3-dipolar cycloaddition between *click*ECM azides and fluorophore-conjugated alkyne groups. Differential interference contrast (DIC) images ensured the proper adjustment of the focal plane during image acquisition. (*click*)ECM structures were visible in the DIC images (lower row images) as filigree structures. Azides – in case of the *click*ECM – were homogeneously distributed over the entire ECM structure whereas no signal was detected for the unmodified ECM. Scale bar s: 400 µm.

Please do not adjust margins

Please do not adjust margins

2.2. Polarisation Microscopy

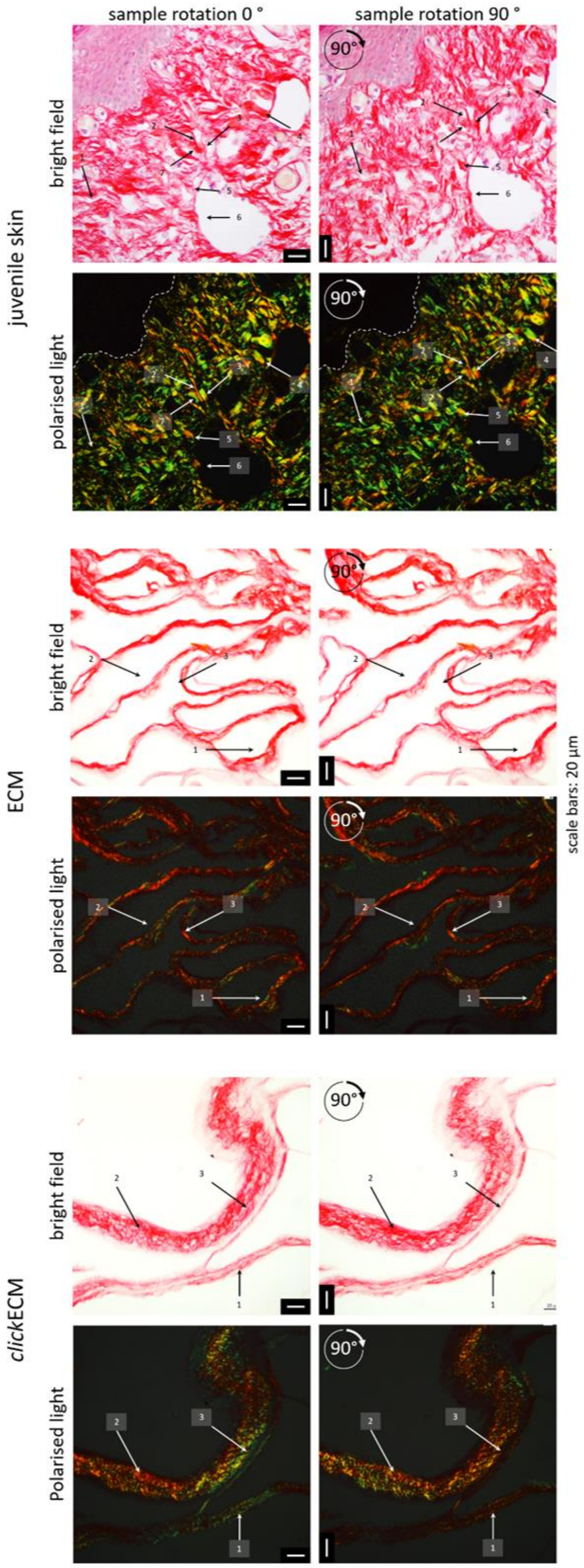

Figure S 2: Bright field and polarisation microscopic images of thin sections from juvenile skin, unmodified ECM, and azide-modified *click*ECM stained by Picro Sirius Red. Images were acquired before and after the microscope stage was rotated by 90°. White arrows point to the same positions and indicate the inversion of colours. Thin sections of a juvenile skin served as control to ensure that the detected effects are not just observed for the delicate (*click*)ECM structures but also for dense structures like skin. Scale bars: 20 µm.

This journal is © The Royal Society of Chemistry 2020

Please do not adjust margins

Please do not adjust margins

3. Quantitative Assessment of *click*ECM Composition

3.1. Quantification of the total Protein Content within (*click*)ECM

3.1.1. Light Microscopic Analysis of a Colorimetric Assay Reaction Solutions

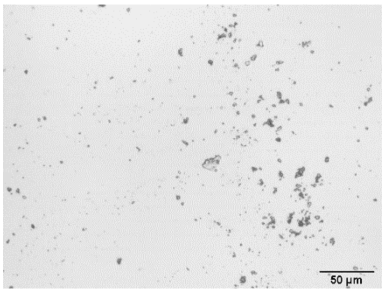

Figure S 3: Representative light microscopic image of a BCA assay reaction solution. Insoluble (*click*)ECM fragments were still visible as such. Scale bar: 50 µm.

This journal is © The Royal Society of Chemistry 2020

Please do not adjust margins

Please do not adjust margins

3.1.2. Calculation of Nitrogen-to-Protein Conversion Factors Based on ECM-Specific Proteins

For the estimation of the total protein content, the specific amino acid sequences (polypeptide chains in the mature protein following processing or proteolytic cleavage) for the five ECM-specific proteins collagen type I, III, IV, fibronectin, and laminin were downloaded from https://www.uniprot.org/ [1] (Table S 1). Hydroxylated proline and lysine residues were taken into account as given in the database.

Table S 1: Entry names and numbers from the five ECM-specific proteins including the specific alpha chains (collagens) as well as the alpha, beta and gamma subunits (laminin) from https://www.uniprot.org/ [1].

protein		entry Name	entry
collagen	type I	CO1A1_HUMAN	P02452
		CO1A2_HUMAN	P08123
	type III	CO3A1_HUMAN	P02461
	type IV	CO4A1_HUMAN	P02462
		CO4A2_HUMAN	P08572
		CO4A3_HUMAN	Q01955
		CO4A4_HUMAN	P53420
		CO4A5_HUMAN	P29400
		CO4A6_HUMAN	Q14031
fibronectin		FINC_HUMAN	P02751
laminin	subunit alpha	LAMA1_HUMAN	P25391
		LAMA2_HUMAN	P24043
		LAMA3_HUMAN	Q16787
		LAMA4_HUMAN	Q16363
		LAMA5_HUMAN	O15230
	subunit beta	LAMB1_HUMAN	P07942
		LAMB2_HUMAN	P55268
		LAMB3_HUMAN	Q13751
	subunit gamma	LAMC1_HUMAN	P11047
		LAMC2_HUMAN	Q13753
		LAMC3_HUMAN	Q9Y6N6

The total nitrogen content $\%N_{total}$ of the proteins was calculated by ($\%N_{AA}$ = nitrogen content of protein-bound amino acid residue, M_{AA} = molar mass of protein-bound amino acid residue, n_{AA} = abundance or molar fraction of amino acid residues in the protein):

$$\%N_{total} = \frac{\sum \%N_{AA} \cdot M_{AA} \cdot n_{AA}}{\sum M_{AA} \cdot n_{AA}} \qquad (1)$$

The nitrogen-to-protein conversion factors were then given by $\frac{1}{\%N_{total}}$.

Please do not adjust margins

Please do not adjust margins

3.1.3. Overview Over Reviewed Literature for the Calculation of Specific Nitrogen-to-Protein and Hydroxyproline-to-Collagen conversion factors

Table S2: Collection of reviewed literature on different tissues and individual ECM-specific biomolecules from different species which were calculated from the amino acid sequences of the mature ECM proteins retrieved from the universal protein database (UniProt[1]) for the calculation of specific nitrogen-to-protein conversion factors as well as specific hydroxyproline (HP)-to-collagen conversion factors. Conversion factors highlighted in yellow were used for the conversion of the nitrogen contents into total protein and conversion factors highlighted in green were used for the conversion of the HP into collagen contents determined in this study (n/s = not further specified; - = not given).

sample	species	nitrogen-to-protein conversion factor	HP-to-collagen conversion factor	reference	analytical method
**tissue**	n/s	-	0.140	Etherington and Sims[2]	-
	mammal	-	0.140	Hofman *et al.*[3]	(factor adapted from Piez and Gross[4]) amino acid analysis
**skin**	human	**5.45**	**0.135**	Bornstein and Piez[5]	amino acid analysis
		**5.56**	**0.133**	Miyahara *et al.*[6]	amino acid analysis
		0.141	-	Smith *et al.*[7]	(factor adapted from Eastoe[8]) resin chromatography ox-hide gelatin
	pig	0.100 – 0.140 (mean 0.125)	-	Edwards and O'Brien Jr.[9]	(factor adapted from Eastoe[8]) amino acid analysis
**gelatin**	pig skin (gelatin type A)	5.25	0.134	Claaßen *et al.*[10]	amino acid analysis
	bovine bone (gelatin type B)	5.45	0.147	Sewald *et al.*[11]	amino acid analysis
**COL I**	mammal	-	**0.135**	Capella-Monsonis *et al.*[12]	n/s
	human	**5.25**	0.104	UniProt[1]	-
	bovine	5.34	0.122		
	rat (skin)	-	0.131	Etherington and Sims[2]	(factor adapted from Stoltz *et al.*[13]) amino acid analysis
	bovine (skin)	5.46	0.143	Bailey and Sims[14]	amino acid analysis
**COL III**	human	**5.31**	0.195	UniProt[1]	-
	bovine	5.16	0.000		
	human (infant dermis)	5.48	**0.180**	Etherington and Sims[2]	(factor adapted from Chung and Miller[15]) amino acid analysis
	bovine (skin)	5.45	0.176	Bailey and Sims[14]	amino acid analysis
**COL IV**	human (α1(IV))	**5.69**	0.014	UniProt[1]	-
	bovine (α1(IV))	5.65	0.012		
	bovine (α2(IV))	6.04	0.000		
	bovine (α3(IV))	5.79	0.001		
	bovine (α4(IV))	5.65	0.000		
	bovine (lens capsule)	-	0.166	Etherington and Sims[2]	(factor adapted from Kefalides[16]) amino acid analysis
	human (placental basement membrane)	6.08	0.197	Glanville *et al.*[17]	amino acid analysis
**fibronectin**	human	**5.88**	0.000	UniProt[1]	-
**laminin**	human (average)	**5.66**	0.000	UniProt[1]	-

This journal is © The Royal Society of Chemistry 2020

Please do not adjust margins

Please do not adjust margins

3.1.4. Statistical Analysis of the calculated Protein Concentrations within (*click*)ECM

Table S3: Analysis of the statistically significant differences between the protein contents of (*click*)ECM derived from the individual nitrogen-to-protein conversion factors for the ECM-specific biomolecules collagen type I (COL I), collagen type III (COL III), collagen type IV (COL IV), laminin (LN), fibronectin (FN). Furthermore, Jones factor for gelatin[18, 19] as well as calculated factors derived from the studies from Bornstein and Piez[5] and Miyahara *et al.*[6] on human infant skin gelatin were used.

		COL I	COL III	skin[5]	gelatin[18, 19]	COL IV	skin[6]	LN	FN
		5.25	5.31	5.45	5.55	5.69	5.65	5.66	5.88
**COL I**	5.25								
**COL III**	5.31	n. s.							
**skin[5]**	5.45	n. s.	n. s.						
**gelatin[18, 19]**	5.55	*	n. s.	n. s.					
**COL IV**	5.69	**	**	n. s.	n. s.				
**skin[6]**	5.65	**	*	n. s.	n. s.	n. s.			
**LN**	5.66	**	*	n. s.	n. s.	n. s.	n. s.		
**FN**	5.88	***	***	**	**	n. s.	n. s.	n. s.	

p-values lower than α = 0.05 (∗), α = 0.01 (∗∗), or α = 0.001 (∗∗∗) were defined as statistically significant. n. s. = difference statistically not significant

3.2. Quantification of the Collagen Content Within (*click*)ECM via the Hydroxyproline (HP) Concentration

From amino acid compositions, the total hydroxyproline contents $\%HP_{total}$ were calculated as (M_{HP} = molar mass of protein-bound hydroxyproline residues, n_{HP} = abundance or molar fraction of protein-bound hydroxyproline residues, M_{AA} = molar mass of protein-bound amino acid residue, n_{AA} = abundance or molar fraction of amino acid residues in the protein):

$$\%HP_{total} = \frac{M_{HP} \cdot n_{HP}}{\sum M_{AA} \cdot n_{AA}} \quad (2)$$

Because the assays determine the free, hydrolysed hydroxyproline, the HP-to-collagen conversion factor R was calculated by Equation (3), where $M_{HP,hydr}$ is the molar mass of free hydroxyproline after hydrolysis:

$$R = \%HP_{total} \cdot \frac{M_{HP,hydr}}{M_{HP}} = \%HP_{total} \cdot \frac{131.13}{113.11} \quad (3)$$

This journal is © The Royal Society of Chemistry 2020

Please do not adjust margins

Please do not adjust margins

4. Supporting Information References

1. U. Consortium, *Nucleic acids research*, 2015, **43**, D204-D212.
2. D. J. Etherington and T. J. Sims, *Journal of the Science of Food and Agriculture*, 1981, **32**, 539-546.
3. K. Hofman, B. Hall, H. Cleaver and S. Marshall, *Analytical biochemistry*, 2011, **417**, 289-291.
4. K. A. Piez and J. Gross, *Journal of Biological Chemistry*, 1960, **235**, 995-998.
5. P. Bornstein and K. A. Piez, *The Journal of clinical investigation*, 1964, **43**, 1813-1823.
6. T. Miyahara, S. Shiozawa and A. Murai, *Journal of gerontology*, 1978, **33**, 498-503.
7. J. G. Smith, E. A. Davidson, W. M. Sams and R. D. Clark, *Journal of Investigative Dermatology*, 1962, **39**, 347-350.
8. J. Eastoe, *Biochemical Journal*, 1955, **61**, 589.
9. C. Edwards and W. O'Brien Jr, *Clinica chimica acta*, 1980, **104**, 161-167.
10. C. Claaßen, M. H. Claaßen, V. Truffault, L. Sewald, G. E. M. Tovar, K. Borchers and A. Southan, *Biomacromolecules*, 2018, **19**, 42-52.
11. L. Sewald, C. Claassen, T. Gotz, M. H. Claassen, V. Truffault, G. E. M. Tovar, A. Southan and K. Borchers, *Macromolecular bioscience*, 2018, **18**, e1800168.
12. H. Capella-Monsonis, J. Q. Coentro, V. Graceffa, Z. N. Wu and D. I. Zeugolis, *Nature Protocols*, 2018, **13**, 507-529.
13. M. Stoltz, H. Furthmayr and R. Timpl, *Biochimica et Biophysica Acta (BBA)-Protein Structure*, 1973, **310**, 461-468.
14. A. J. Bailey and T. J. Sims, *Biochemical Journal*, 1976, **153**, 211-215.
15. E. Chung and E. J. Miller, *Science*, 1974, **183**, 1200-1201.
16. N. A. Kefalides, *Biochemical and biophysical research communications*, 1972, **47**, 1151-1158.
17. R. W. Glanville, A. Rauter and P. P. Fietzek, *European journal of biochemistry*, 1979, **95**, 383-389.
18. F. Mariotti, D. Tome and P. P. Mirand, *Critical Reviews in Food Science and Nutrition*, 2008, **48**, 177-184.
19. D. B. Jones, *Factors for converting percentages of nitrogen in foods and feeds into percentages of proteins*, US Department of Agriculture, Washington, D. C., 1931.

This journal is © The Royal Society of Chemistry 2020

Please do not adjust margins
